# Transactions of Medical Associations

**Published:** 1852-11

**Authors:** 


### *Transactions of Medical Associations.*

*Transactions of the Medical Association of Southern Central New York, at the Annual Meeting, held at Owego, June,* 1852.

The Transactions of this Association for the present year, embrace: 1st. The annual address of the president, Dr. James H. Jerome, of Trumansburg. The subject is Quackery, which, considering that the theme is rather hackneyed, is well handled. 2d. The vital statistics of Cortland county, by Dr. Caleb Green. This report contains meteorological tables for the year, and a succinct account of the prevailing diseases during that period. Similar reports, from different parts of the county, would supply data for important deductions. 3d. Vital Statistics of Owego, by L. H. Allan, M. D. 4th. Abstract of report of committee on Surgery for Cortland Co., by F. Hyde, M. D., chairman. This, like former reports by the same author, is an interesting paper. 5th. Surgical report of Tioga Co., by R. O. Crandall, M. D. 6th. Report of committee on Surgery for the county of Broome, by Geo. Bun, M. D., chairman. This report contains an interesting case of fracture of vertebræ.

In addition to the foregoing, the volume contains reports of cases by Drs. S. E. Clark, N. R. Derby, T. H. Squire, J. H. Jerome, and Wm. C. Wey.

Our readers will perceive by this brief notice, that the Association of Southern Central New York, is sustained with unabated zeal. The example of our brethren in the counties over which the association extends should excite an effort to institute similar organizations in other districts throughout the empire state.

*Transactions of the Medical Association of the State of Missouri, at its Second Annual Meeting, St. Louis, vol.* II, 1852.

This is a volume of 116 pages, embracing communications and reports on various subjects. The association is of recent formation, but the volume before us gives promise of success and usefulness. Dr. W. M. McPheeters, of St. Louis, is president of the association.

*Transactions of the Illinois State Medical Society for the year* 1852.

This makes a volume of 94 pages, and contains some valuable papers, among which is an interesting summary of the contributions to practical medicine, by Prof. N. S. Davis, chairman of the committee. The society, although of recent formation, is in a flourishing condition.

*The Transactions of the Third Annual Meeting of the Medical Society of the State of Georgia, held in the city of Augusta, April,* 1852.

This is a volume of 100 pages. Among other papers of interest, it embraces a report of twenty-five cases of urinary calculus, in twenty-three of which the bi-lateral operation was performed, by Prof. Paul F. Eve; observations on the use of certain new remedies, by Prof. L. A. Duges, and an able address by H. F. Campbell, first vice-president of the society, on "the difficulties and the privileges of the medical profession."

The several volumes which we have briefly noticed, furnish evidence of an increasing disposition among members of the profession in different sections of the republic, to secure the benefits of union for professional and scientific objects, and they show a degree of activity and spirit pervading the profession which it may be confidently expected will lead to happy results.

